# Impact of Artificial Intelligence-Enhanced Insertable Cardiac Monitors on Device Clinic Workflow and Resource Utilization

**DOI:** 10.1016/j.jacadv.2025.101656

**Published:** 2025-03-19

**Authors:** Aashish Katapadi, Nikhila Chelikam, Sarah Rosemas, Lucas Higuera, Ilyas Colombowala, Shanti Bansal, Douglas Darden, Naga Venkata K. Pothenini, Scott Koerber, Rangarao Tummala, Peter Park, Rakesh Gopinathannair, Dhanunjaya Lakkireddy, Rajesh Kabra

**Affiliations:** a Kansas City Heart Rhythm Institute, Overland Park, Kansas, USA; b Medtronic Cardiac Rhythm Management, Mounds View, Minnesota, USA; c Houston Electrophysiology Associates, Houston, Texas, USA; d Houston Heart Rhythm, Houston, Texas, USA; e Octagos Health, Houston, Texas, USA

**Keywords:** AI, artificial intelligence, cardiac arrhythmias, health economics, insertable cardiac monitors, remote monitoring

## Abstract

**Background:**

Insertable cardiac monitors (ICMs) are essential for managing arrhythmias but often generate large numbers of transmissions and false alerts. Integrating artificial intelligence (AI) as part of the ICM workflow can reduce this burden. However, its impact on clinic workflow and resource utilization must be better understood.

**Objectives:**

The aim of the study was to assess the impact of AI-enhanced ICMs on clinic workflow and resource utilization.

**Methods:**

A cross-sectional analysis was conducted using real-world, deidentified ICM remote monitoring data from Octagos Health, which included 140 U.S. device clinics between July 2022 and April 2024. Nonactionable alerts (NAAs) were defined as false or repetitive alerts transmitted on the remote monitoring platforms but dismissed by device technicians and not forwarded to clinicians for review. We compared NAAs generated by AI-enhanced vs non-AI-enhanced ICMs and estimated associated staffing hours, resources, and costs extrapolated for a clinic managing 600 ICM patients.

**Results:**

Among 19,320 patients (mean age: 69 ± 13.5 years; 47.3% male), 68% had non-AI-enhanced ICMs, and 32% had AI-enhanced ICMs. The mean annual NAA volume per 600-ICM clinic was 5,078 for non-AI-enhanced ICMs and 2,110 for AI-enhanced ICMs, resulting in 559 fewer staffing hours (956 vs 397 hours; 95% CI: 513-605 hours; *P* value < 0.001) and $29,470 in annual savings ($20,929 vs $50,399; 95% CI: $27,035-$31,904; *P* value < 0.001).

**Conclusions:**

Compared to non-AI-enhanced ICMs, AI-enhanced ICMs significantly reduce NAAs, leading to a projected decrease in clinic workload and associated costs, potentially improving workflow and health care efficiency.

Insertable cardiac monitors (ICMs) play a significant role in identifying and managing heart rhythm disorders.^1^ Over the years, these devices have undergone significant technological advances, including enhanced accuracy for arrhythmia diagnosis, smaller sizes with percutaneous implantation, and remote transmissions for expeditious arrhythmia diagnosis to improve patient outcomes. As a result, their use has increased exponentially in arrhythmia diagnosis and management. However, the data deluge from remote monitoring of ICMs and subsequent clinically irrelevant, false, or repetitive, nonactionable alerts (NAAs) poses a significant challenge to device clinics.^2^

Integrating artificial intelligence (AI) technologies into ICM workflow can alleviate the burden of NAAs. Manufacturers have recently begun developing proprietary algorithms employing various levels of AI to accurately filter transmissions and increase an ICM's positive predictive value.^3^^,^^4^ A few ICM algorithms currently incorporate AI, one being the AccuRhythm AI algorithms for the LINQ II ICMs (Medtronic). The impact of AI algorithms on device clinic workflow and resource utilization remains poorly described. In the current study, we explored whether manufacturer-specific AI algorithms affect the burden of NAAs from remote monitoring data of ICMs. We further utilized previously reported workflow models to quantify the potential impact on clinic work hours and the projected financial savings.

## Methods

### Study design and patient selection

We performed a cross-sectional study of real-world, deidentified remote cardiac monitoring data from ICMs collected between July 2022 and April 2024 across 140 device clinics in the United States. Monitored patients included those with (AI-enhanced) and without (non-AI-enhanced) performance-optimized deep learning AI ICMs. The AI-enhanced cohort consisted of patients with the Medtronic LINQ II ICM, and the non-AI-enhanced cohort consisted of all other ICM models by Medtronic and other manufacturers, including Boston Scientific Corporation, Abbott Laboratories, and Biotronik. Primary outcomes of interest included reductions in NAAs, clinic workload, and associated staffing costs of reviewing NAAs between the 2 groups during the 21-month follow-up period. The study was reviewed by the Western Institutional Review Board and exempt from oversight, and no patient consent was required due to the deidentified nature of the study.

### AI-enhanced algorithms

The AccuRhythm AI consists of a suite of cloud-based, multilayered convolutional neural network algorithms that are applied to both device-detected pauses and atrial fibrillation episodes, where those tagged as false by the AI are then filtered from clinician viewing. The algorithms were trained using supervised learning from a database of >1 million electrocardiograms adjudicated by a panel of experts. Their performance was tested on 2 independent validation datasets—1,856 ICM-detected atrial fibrillation episodes from 955 patients and 2,766 ICM-detected pause episodes from 382 patients—and reduced false alerts by 88.2% and 97.4%, while retaining 99.2% and 100.0% of true alerts for each arrhythmia, respectively.

Another ICM incorporating AI is the SmartECG algorithm for the BIOMONITOR IV ICM (Biotronik). The algorithm provides AI labeling of inappropriate device-detected episodes to aid clinical review and triaging. Unlike the AccuRhythm AI, which filters out NAA from remote monitoring, the SmartECG does not filter these episodes from the clinician's view. As such, for this characterization of the workflow burden reduction of AI-eliminated NAAs, the SmartECG algorithm was not included within the AI-enhanced cohort. Likewise, the Reveal LINQ devices did not yet have AccuRhythm AI algorithm capabilities at the time of this analysis. Thus, the only device defined within the AI-enhanced cohort was the LINQ II ICM with AccuRhythm AI.

### Device transmissions

Device transmissions were adjudicated by Octagos Health—a cloud-based ICM data processing platform that utilizes AI-based software automation. Transmissions routed through the Octagos Health platform are either dismissed or sent for further clinical review; alert-based transmissions are sent to the clinician only if clinically actionable, based on alert type or information in the medical record. Up to 15% of all these transmissions are reviewed at random by an independent, board-certified electrophysiologist consulted by Octagos Health to ensure accuracy. Device transmissions were scheduled as per the 2023 Heart Rhythm Society consensus statement on the management of remote device clinics.^5^ Total transmissions were stratified by the device manufacturer and extrapolated to a representative clinic supporting 600 ICM patients, which was chosen to represent a medium- to large-sized clinic. NAAs were defined as transmissions processed by Octagos Health but not sent to clinicians. These included known arrhythmias, repetitive alerts for which the patient was already on the appropriate medication, or false positive alerts.

### Staffing and cost extrapolation

The mean NAA volumes scaled to a 600-ICM clinic were calculated by cohort and device manufacturer and then extrapolated into staffing hours and associated clinic costs spent reviewing the alerts. Clinic workload hours associated with NAAs were calculated using data from published literature, which estimated that 11.3 minutes of total staff time was spent per NAA.^6^ To calculate staffing cost per hour, the distribution of clinic staff types reviewing ICM alerts was assumed to be 1 registered nurse, 2 medical assistants, and 1 advanced practice provider, based on the 2023 Heart Rhythm Society Consensus statement.^5^ Associated staffing costs for each staff member were calculated based on the U.S. Bureau of Labor Statistics national average wage estimates for 2022, as well as the cost of benefits, which accounts for 29.4% of total compensation in health care industry employees.^7^^,^^8^ Overall, the weighted average cost per hour of staff member time was $52.71.

### Statistical analysis

NAA volumes, projected workload, and associated costs were compared across AI-enhanced vs non-AI-enhanced cohorts and device manufacturers. Generalized linear models were used to calculate standard errors and CIs of the percent differences in staffing hours and cost savings. Specifically, percent differences were calculated with a generalized linear model with a log link and gamma distribution, and absolute differences were calculated with a generalized linear model with an identity link and a normal distribution; models include either a binary flag for AI-enhanced vs non-AI-enhanced or a categorical variable by the manufacturer as the regressor of interest. No additional covariates were included in any model. A *P* value <0.05 was considered statistically significant, and estimands are provided with 95% CIs. All statistical analyses were performed using SAS software version 9.4 (SAS Institute Inc).

## Results

### Study data

A total of 19,320 patients monitored by ICMs were included in the study. The mean age was 69 ± 13.5 years, and 47.3% of patients were male. A total of 6,109 patients (31.6%) had an AI-enhanced ICM, and 13,211 patients (68.4%) had non-AI-enhanced ICMs. Table 1 categorizes them by the manufacturer. A total of 467,746 transmissions were evaluated during the study period.

**Table 1:** Distribution of Patients

Manufacturer	N (%)
**AI-enhanced ICM**	
Medtronic LINQ II ICM	6,109 (31.6%)
Non-AI-enhanced ICMs	
Other Medtronic	4,606 (23.8%)
Boston Scientific Corporation	2,735 (14.2%)
Abbott Laboratories	3,312 (17.1%)
Biotronik	2,558 (13.2%)
Total	13,211 (68.4%)

The distribution of patients in the AI- and non-AI-enhanced cohorts in 13,211 patients.

AI = artificial intelligence; ICM = insertable cardiac monitor.

### Total annualized impact of AI-enhanced ICM on NAAs

**Figure 1:**
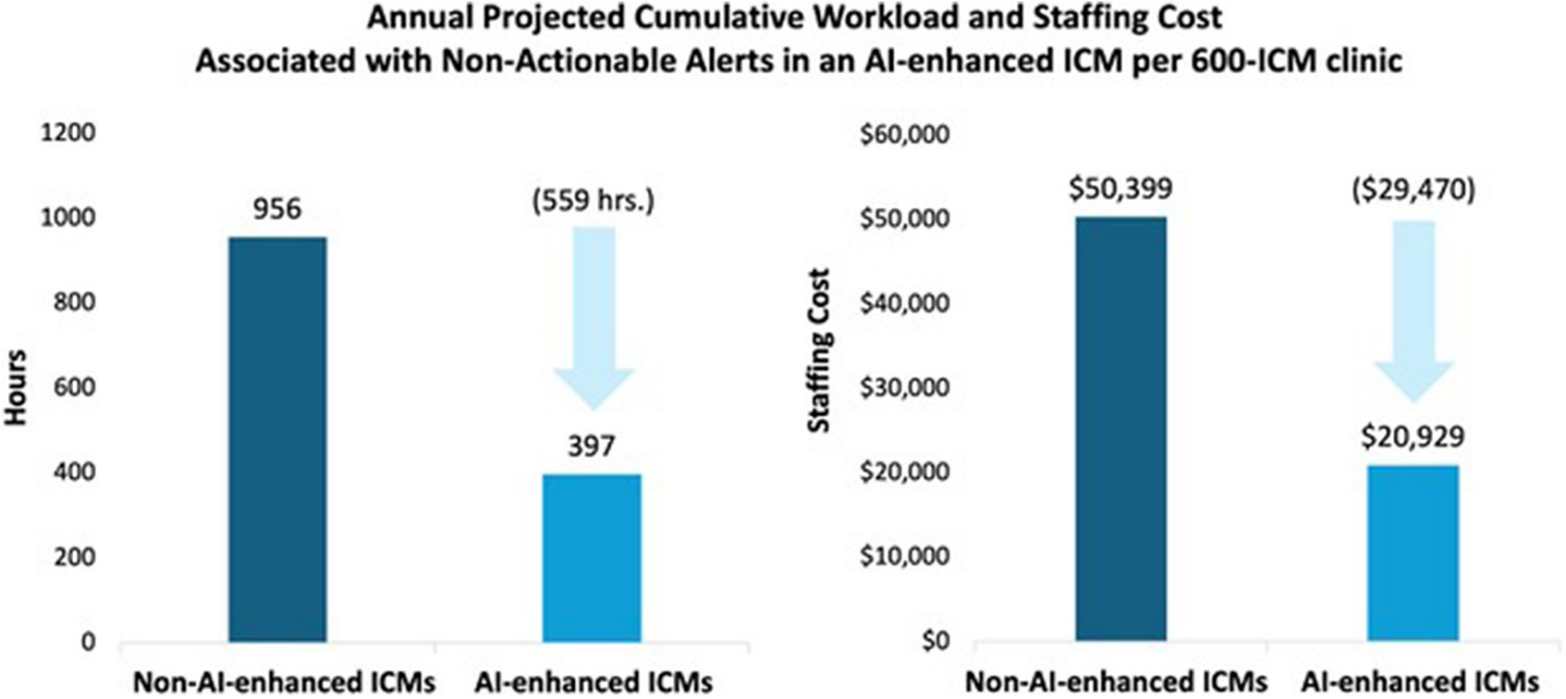
Annualized Workload and Staffing Cost The projected workload and staffing costs were associated with a 58.5% reduction in ICM NAA volume per 600-ICM clinic over 12 months. AI = artificial intelligence; ICM = insertable cardiac monitor; NAA = nonactionable alert.

The mean NAA annual volume per 600-ICM clinic was less in the AI-enhanced cohort (2,110) than in the non-AI-enhanced (5,078) cohort (Table 2). This resulted in a 58.5% reduction in annual NAAs when utilizing an AI-enhanced ICM (95% CI: 57% to 60%, *P* < 0.001). This significant reduction in NAAs resulted in a projected 559-hour annual difference in estimated staffing time compared to non-Al-enhanced ICMs (397 vs 956 hours; 95% CI: 513-605; *P* < 0.001), which equated to $29,470 in staffing costs (Figure 1; $20,929 vs $50,399; 95% CI: $27,035-$31,904; *P* < 0.001) (Central Illustration).

**Table 2:** Annual NAA Volumes and Clinic Work Hours and Cost

Manufacturer	Annual NAAs	Annual NAAs per Patient	Projected Annual Hours Reviewing NAAs	Projected Annual Costs Reviewing NAAs
**AI-enhanced ICM**				
Medtronic LINQ II ICM	2,110	3.5	397	$20,929
Non-AI-enhanced ICMs				
Other Medtronic	4,295	7.2	809	$42,624
Boston Scientific Corporation	7,907	13.2	1,489	$78,479
Abbott Laboratories	4,481	7.5	844	$44,477
Biotronik	4,232	7.1	797	$42,000
Weighted mean	**5,078**	**8.7**	**956**	**$50,399**
Reduction in NAA in AI-enhanced vs non-AI-enhanced ICMs^a^	58.5% (CI: 57%-60%), *P* value = <0.001			

The associated NAA volumes, work hours, and costs in AI-enhanced vs non-AI-enhanced ICMs per 600-ICM clinic. In bold: weighted NAA averages, hours and costs.

AI = artificial intelligence; ICM = insertable cardiac monitor; NAA = nonactionable alert.

a Difference was calculated with a generalized linear model with a log link and gamma distribution.

**Central Illustration:**
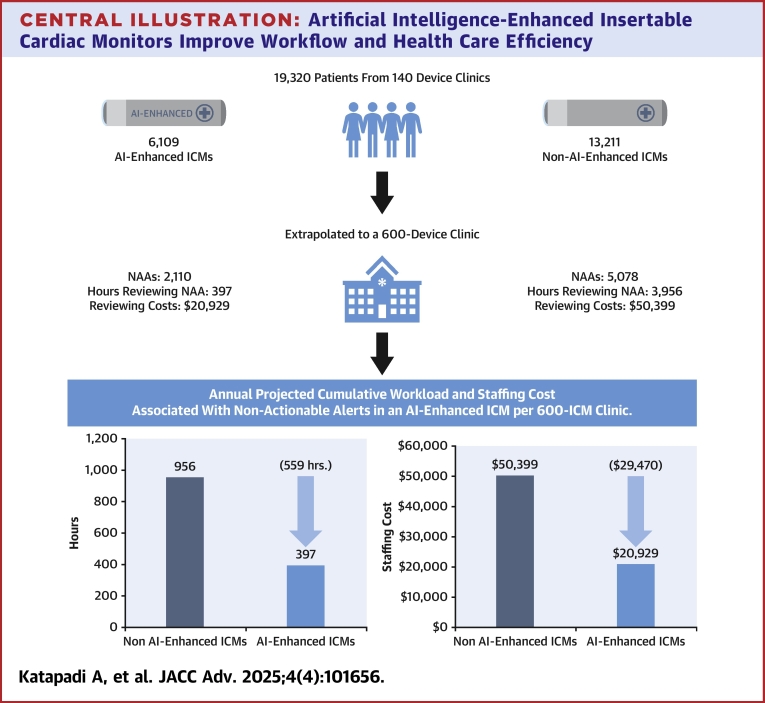
Artificial Intelligence-Enhanced Insertable Cardiac Monitors Improve Workflow and Health Care Efficiency We extrapolated data from 6,109 AI-enhanced implantable cardiac monitors out of 19,320 patients and 140 device clinics to a 600-device clinic. This revealed an improvement of 559 hours and $29,470 in clinic staffing time and cost. AI = artificial intelligence; ICM = insertable cardiac monitor; NAA = nonactionable alert.

### Annualized clinic workload reduction using AI-enhanced ICMs by vendor

The total reduction in annual NAA volume by utilizing an AI-enhanced compared to various non-AI-enhanced ICMs ranged from 50.1% to 73.3%. This led to an estimated impact of 400 to 1,092 hours in associated staffing time, equating to $21,058 to $57,533 in associated clinic costs annually (Table 3).

**Table 3:** Reduction in NAA, Hours, and Costs Using AI-Enhanced ICM in a 600-ICM Clinic^a^

Manufacturer Comparison	% Reduction in NAA	Projected Reduction in Hours Reviewing NAA	Projected Reduction in Cost Reviewing NAA
Medtronic AI-enhanced vs Medtronic non-AI-enhanced	50.1% (CI: 49%-53%)^a^	412 (CI: 355-469)^a^	$21,723 (CI: $18,687-$24,760)^a^
Medtronic AI vs Boston Scientific Corporation	73.3% (CI: 72%-75%)^a^	1,092 (CI: 1,024-1,160)^a^	$57,533 (CI: $53,953-$61,6113)^a^
Medtronic AI vs Abbott Laboratories	52.9% (CI: 50%-55%)^a^	447 (CI: 383-511)^a^	$23,563 (CI: $20,205-$26,921)^a^
Medtronic AI vs Biotronik	50.1% (CI: 47%-53%)^a^	400 (CI: 330-469)^a^	$21,085 (CI: $17,393-$24,722)^a^

Utilizing AI-enhanced ICMs significantly reduced the associated clinical burden compared to all other non-AI-enhanced ICMs.

Abbreviations as in Table 2.

a All CIs were calculated at the 95% significant level with a *P* value < 0.001.

### Total impact of AI-enhanced ICM on long-term follow-up

**Figure 2:**
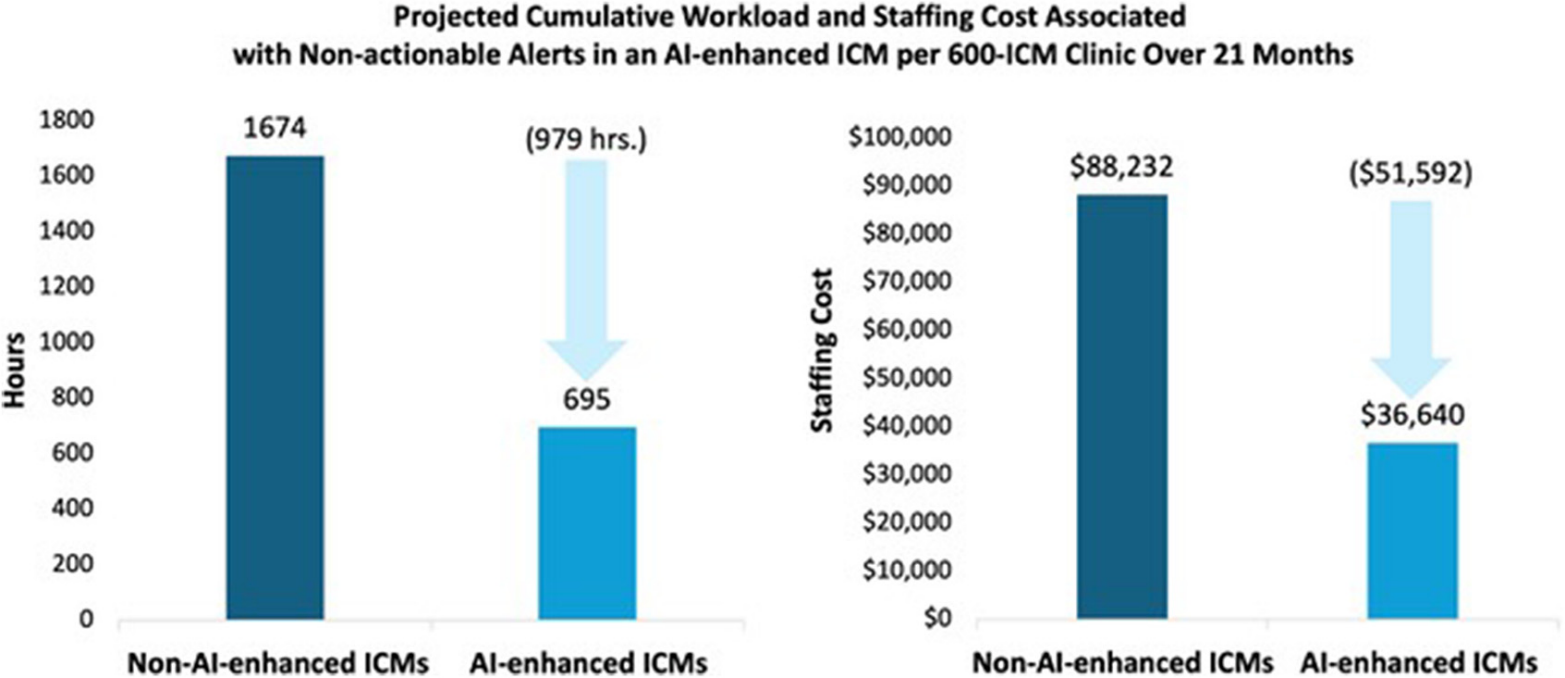
Long-Term Workload and Staffing Cost The projected workload and staffing costs were also associated with reduced NAA volume per 600-ICM clinic over 21 months. AI = artificial intelligence; ICM = insertable cardiac monitor; NAA = nonactionable alert.

During the full follow-up period of 21 months, the mean total NAA volume per 600-ICM clinic in the AI-enhanced cohort was 3,693 compared with 8,889 in the non-AI-enhanced cohort, equivalent to a 58.5% reduction in NAAs by utilizing AI-enhanced ICMs (95% CI: 57% to 60%, *P* < 0.001). This significant reduction resulted in a projected 979-hour difference in estimated staffing time (695 vs 1,674 hours; 95% CI: 898-1,059; *P* < 0.001), which equated to a $51,592 improvement in staffing costs (Figure 2; $36,640 vs $88,232; 95% CI: $47,330-$55,835; *P* < 0.001) during the full 21-month follow-up period.

## Discussion

This study demonstrates that AI-enhanced ICMs are associated with a significant reduction in the volume of NAAs compared to non-AI-enhanced ICMs (Central Illustration). Using a time and motion analysis, we further project that AI-enhanced ICMs will significantly reduce the clinic workload and health care costs associated with managing ICMs.

The clinical impacts of ICMs are well known and include earlier detection of arrhythmias, resulting in earlier rhythm control, greater arrhythmia freedom, and fewer complications.^9^ Yet, the associated data deluge of these devices remains a significant challenge. Several studies have revealed that the vast majority of remote monitoring transmissions are false positives.^10^ AI has been shown to increase classification accuracy by up to 95% and reduce false positives by up to 98%.^3^^,^^11^^,^^12^ Recognizing and stratifying transmissions can further improve the burden of data, but only a few studies have done so. A study by O'Shea et al^13^ concluded that 30.3% of 41,454 ICM alerts required no further action. Similarly, another study reported that 33.8% of 79,504 ICM alerts required no further action.^2^ However, the impact of NAAs on clinical time allocation and workflow remained poorly studied.

Time and motion studies have long been regarded as the most reliable analysis method for this purpose.^14^^,^^15^ A prior study by Afzal et al^16^ estimated the average personnel time to adjudicate a remote transmission from ICMs at 15 ± 6 minutes. Shortly afterward, Seiler et al^6^ estimated the average staff time per ICM transmission at 11.3 and 17.33 minutes for nonactionable and actionable alerts, respectively. Utilizing these results, we demonstrate that using AI-enhanced ICMs decreases the number of annual NAAs by 58.5% and the annual clinical staff time associated with reviewing these alerts by 559 hours in a 600-ICM device clinic. This equates to a projected annual cost savings of $29,470 in staffing expenses.

As the adoption and integration of AI-based algorithms at various levels into the ICM workflow continue to evolve, this study emphasizes the positive impact on patient care. Furthermore, implementing this approach across different aspects of data flow, with each layer focusing on its unique challenges, can incrementally but substantially improve clinical efficiency. We demonstrate that employing validated AI technology to assess ICM-detected episodes decreased NAAs, thereby reducing clinic workload and the associated staffing expenses. These operational efficiencies allow more time for critical patient care activities. Although this study focuses only on 1 AI-enhanced ICM, this benefit may be observed across all future AI-enabled ICMs. However, the performance of these algorithms will need to be evaluated in clinical studies, as not all AI algorithms are equal, too much layering may result in loss of sensitivity, and there are no data on how individual AI layers perform prealgorithm and postalgorithm implementations.

### Study Limitations

This study has a few limitations that affect its interpretation. This is an observational study of information provided by Octagos Health—a single remote monitoring company. Although conclusions may not be generalizable, the Octagos Health dataset is large and representative of the real world. The study measurements are also estimated based on presumed typical device clinic workflows and externally published data. As a result, the extrapolated data may again not be generalizable to all clinics. Nevertheless, this study is the first step toward studying the effects of AI in the ICM clinic workflow. Further research, including randomized control trials and prospective studies, is needed to evaluate the impact and role of AI in cardiac monitoring.

## Conclusions

AI-enhanced ICMs significantly reduce device clinics' workload by reducing the burden of NAAs. This could decrease health care costs and improve patient care by saving clinic time to more efficiently address actionable alerts. Increased adoption of AI-enhanced devices can further improve operational workflow, timely and precise patient diagnosis, and overall health care efficiency.

## Funding support and author disclosures

The open access fee was funded by Medtronic. Drs Colombowala and Bansal are shareholders of Octagos Health. Dr Pothineni is a consultant for Medtronic and Biosense Webster. Dr Gopinathannair is a consultant for Boston Scientific. Dr Lakkireddy is a consultant for Abbott Vascular, Biotronik, BioSense Webster, Medtronic, Boston Scientific, Atricure, Acutus, and Northeast Scientific. Dr Kabra is a consultant for Volta Medical. Ms Rosemas and Mr Higuera are employees and shareholders of Medtronic. All other authors have reported that they have no relationships relevant to the contents of this paper to disclose.
